# Efficacy of intravenous plus intrathecal/intracerebral ventricle injection of polymyxin B for post-neurosurgical intracranial infections due to MDR/XDR *Acinectobacter baumannii*: a retrospective cohort study

**DOI:** 10.1186/s13756-018-0305-5

**Published:** 2018-01-19

**Authors:** Sijun Pan, Xiaofang Huang, Yesong Wang, Li Li, Changyun Zhao, Zhongxiang Yao, Wei Cui, Gensheng Zhang

**Affiliations:** 1 Department of Critical Care Medicine, Second Affiliated Hospital, Zhejiang University School of Medicine, Hangzhou, Zhejiang 310009 People’s Republic of China; 2 Department of Critical Care Medicine, Anji County People’s Hospital, Huzhou, Zhejiang Province 313300 China; 3 Department of Critical Care Medicine, Zhejiang Hospital, Hangzhou, 310013 China

**Keywords:** *Acinetobacter Baumannii*, Polymyxin B, Intrathecal injection, Intracerebral ventricle injection, Multidrug resistance

## Abstract

**Background:**

Post-neurosurgical intracranial infections caused by multidrug-resistant or extensively drug-resistant *Acinetobacter baumannii* are difficult to treat and associated with high mortality. In this study, we analyzed the therapeutic efficacy of intravenous combined with intrathecal/intracerebral ventricle injection of polymyxin B for this type of intracranial infection.

**Methods:**

This retrospective study was conducted from January 2013 to September 2017 at the Second Affiliated Hospital, Zhejiang University School of Medicine (Hangzhou,China) and included 61 cases for which cerebrospinal fluid (CSF) cultures were positive for multidrug-resistant or extensively drug-resistant *A. baumannii* after a neurosurgical operation. Patients treated with intravenous and intrathecal/intracerebral ventricle injection of polymyxin B were assigned to the intrathecal/intracerebral group, and patients treated with other antibiotics without intrathecal/intracerebral injection were assigned to the intravenous group. Data for general information, treatment history, and the results of routine tests and biochemistry indicators in CSF, clinical efficiency, microbiological clearance rate, and the 28-day mortality were collected and analyzed.

**Results:**

The rate of multidrug-resistant or extensively drug-resistant *A. baumannii* infection among patients who experienced an intracranial infection after a neurosurgical operation was 33.64% in our hospital. The isolated *A. baumannii* were resistant to various antibiotics, and most seriously to carbapenems (100.00% resistance rate to imipenem and meropenem), cephalosporins (resistance rates of 98.38% to cefazolin, 100.00% to ceftazidime, 100.00% to cefatriaxone, and 98.39% to cefepime). However, the isolated *A. baumannii* were completely sensitive to polymyxin B (sensitivity rate of 100.00%), followed by tigecycline (60.66%) and amikacin (49.18%). No significant differences in basic clinical data were observed between the two groups. Compared with the intravenous group, the intrathecal/intracerebral group had a significantly lower 28-day mortality (55.26% vs. 8.70%, *P* = 0.01) and higher rates of clinical efficacy and microbiological clearance (95.65% vs. 23.68%, *P* < 0.001; 91.30% vs. 18.42%, *P* < 0.001, respectively).

**Conclusions:**

Intravenous plus intrathecal/intracerebral ventricle injection of polymyxin B is an effective regimen for treating intracranial infections caused by multidrug-resistant or extensively drug-resistant *A. baumannii.*

## Background

Postoperative nervous system infection is a common complication of neurosurgery and accounts for 0.8–7% of intracranial infections [1]. The most common pathogens are Gram-negative *Bacilli* and *Staphylococcus aureus*, but the percentage of post-neurosurgical intracranial infections caused by *Acinetobacter baumannii* is still high at 15–21.74% [2, 3] with a high associated mortality rate ranging from 20 to 40% [4, 5]. A previous study reported a frequency of nosocomial, post-neurosurgical meningitis caused by *A. baumannii* as high as 10.9% with a mortality rate of 33.3% [6]. An urgent clinical problem that has arisen in recent years is the high prevalence of intracranial infections with multidrug-resistant (MDR)/extensively drug-resistant (XDR) *A. baumannii* (MDR/XDR-Ab) due to the widespread use of broad-spectrum antibiotics. Thus, effective treatments for MDR/XDR-Ab intracranial infections are needed.

Although MDR/XDR-Ab is resistant to multiple antibiotics, it is still currently susceptible to polymyxins. The high molecular weight of polymyxins and the existence of the blood–brain barrier have forced the use of "intravenous combined with intrathecal/intracerebral ventricle injection" in order to achieve an effective therapeutic concentration [7, 8]. This method has been used clinically to treat intracranial infections caused by MDR/XDR-Ab, but the outcomes have been described in mostly case reports or case series [9, 10]. To further confirm the efficacy of this treatment strategy and provide more solid evidence, we retrospectively analyzed the effects of intravenous antibiotics without polymyxin B and intravenous plus intrathecal/intraventricular injection of polymyxin B in 61 cases of intracranial infection with MDR/XDR-Ab after neurosurgery.

## Methods

### Patients

**Fig. 1:**
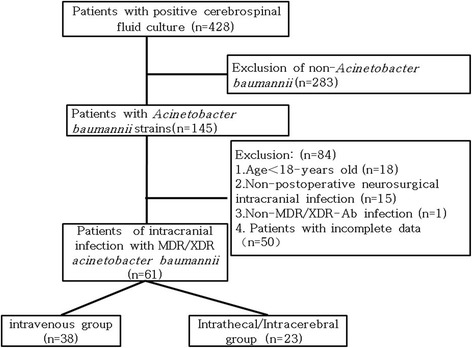
Flowchart of study participant enrollment

This single-center retrospective cohort study was conducted from January 2013 to September 2017 at the Second Affiliated Hospital, Zhejiang University School of Medicine (Hangzhou, China), and consecutive, unselected adult patients (age > 18 years) with a diagnosis of intracranial infection due to MDR/XDR-Ab after a neurosurgery were enrolled. The exclusion criteria were: a poly-microbial result from cerebrospinal fluid (CSF) culture; non-MDR/XDR-Ab intracranial infection; MDR/XDR-Ab intracranial infection not occurring as a complication after neurosurgery; or intracranial colonization due to MDR/XDR-Ab. Patients also were excluded from the study if they were pregnant or had a malignancy outside of the nervous system. Figure 1 outlines the selection of the patients. Sixty-one patients were assessed as eligible for inclusion in this study, including 38 in the intravenous only group and 23 in the intravenous plus intrathecal/intracerebral group. The diagnostic criteria for post-neurosurgical intracranial infection due to MDR/XDR-Ab were as reported [11, 12]: (1) a positive CSF culture for MDR/XDR-Ab. MDR was defined as resistance to at least one agent in three or more antimicrobial categories (such as carbapenems, aminoglycosides, and cephalosporins); XDR was defined as resistance to all other antimicrobial agents, except one or two antimicrobials (such as tigecycline and polymyxins) [13]. The antibiotic susceptibilities were determined using a Vitek 2 compact automated system (bioMerieux, Marcy-l’Etoile, France) or the disk diffusion method according to the Clinical Laboratory Standards Institute (CLSI) criteria in our microbiology laboratory, and the results were interpreted according to the CLSI 2016 criteria [14]. (2) At least two of the following symptoms with no other recognized cause: fever > 38 °C or headache, meningeal signs, or cranial nerve signs. (3) CSF/serum glucose ratio < 0.5, CSF nucleated cells > 10 × 10^6^ /L, or protein level > 0.45 g/L. A positive CSF culture was defined by colonization or contamination if the patient had no clinical symptoms or had normal levels of glucose, nucleated cells and protein [12].

### Treatment protocol

The patients treated with intravenous and intrathecal/intracerebral ventricle injection of polymyxin B (Fresenius Kabi USA) were assigned to the intrathecal/intracerebral group, and patients treated with intravenous antibiotics only were assigned to the intravenous group. In the intrathecal/intracerebral group, 450,000 units per 12 h were administered intravenously and 50,000 units/day were simultaneously administered via the lumbar cistern drainage tube or ventricular drainage tube twice daily [9–11]. The drainage tube was removed and replaced upon diagnosis of infection. The process of intrathecal/intracerebral injection was as follows: we withdrew 2 mL CSF via the tube and discarded it; then we injected 50,000 units/day of polymyxin B; and then we kept the tube closed for 2 h [9, 11]. In the intravenous group, patients were treated with other antibiotics without intrathecal/intracerebral injection.

### Data collection

Demographic characteristics including age, sex, underlying disease, co-morbidities, operation method, co-infections, and liver and kidney function were reviewed, and the acute physiology and chronic health evaluation (APACHE) II score, sequential organ failure assessment (SOFA) score, and the general history of initial antimicrobial use were also recorded. Symptoms of intracranial infection like temperature and meningeal stimulation, routine and biochemistry indicators in CSF, culture results for CSF, the use of antibiotics, and treatment efficacy were also recorded (Table 1). Evaluation of treatment efficacy was based on the above clinical and microbiologic parameters. Clinical efficiency was defined as the disappearance or improvement of symptoms. Microbiological efficiency was defined as disappearance/clearance of *A. baumannii* from three consecutive CSF cultures after treatment. The primary end point of this study was 28-day mortality, and secondary end points were clinical efficiency and microbiological efficiency.

**Table 1:** Baseline characteristics of patients enrolled in the study

Characteristic	ITV group (*n* = 38)	ITV + ITC group (*n* = 23)	*P*
Sex (male) (n, %)	20, 52.63%	10, 43.48%	0.488
Age (years)	53.50 ± 15.17	55.00 ± 15.08	0.761
Primary disease (n, %)			0.091
Cerebral hemorrhage	30, 78.95%	14, 60.87%	
Craniocerebral trauma	4, 10.53%	2, 8.70%	
Benign Intracranial tumor	4, 10.53%	7, 30.43%	
Comorbidities (n, %)			0.833
Diabetes	3, 7.89%	4, 17.39%	
Cardiovascular disease	11, 28.95%	9, 39.13%	
Pulmonary disease	0, 0.00%	1, 4.35%	
Nervous system disease	1, 2.63%	1, 4.35%	
Surgeries (n, %)			0.396
Craniotomy evacuation of hematoma + decompressivecraniectomy	32, 84.21%	21, 91.27%	
Intracranial tumor resection	3, 7.89%	2, 8.70%	
Craniotomy aneurysm clipping	12, 31.58%	9, 39.13%	
Drainage of intracranial hematoma	9, 23.68%	7, 30.43%	
Ventricle peritoneal shunt	6, 15.79%	5, 21.74%	
Lumbar cistern drainage	26, 68.42%	15, 65.22%	
Ommaya reservoir	2, 5.26%	2, 8.70%	
Coinfection (n, %)			0.727
Lung	29, 76.32%	18, 78.26%	
Bloodstream	5, 13.16%	4, 17.39%	
SOFA score	5.34 ± 3.02	5.08 ± 2.23	0.707
APACHE II score	18.55 ± 5.62	17.65 ± 4.90	0.513
Clinical symptoms			
Fever (°C)	39.02 ± 0.53	39.17 ± 0.48	0.256

*SOFA* sequential organ failure assessment, *APACHE* acute physiology and chronic health evaluation. *ITV* intravenous, *ITV + ITC* intrathecal/intracerebral

### Statistical analysis

Statistical analysis was performed with SPSS 19.0 (SPSS, IBM Company, Chicago, IL) software. Continuous variables are presented as mean ± standard deviation if normally distributed, and as median and interquartile range if non-normally distributed. The Student’s t-test was performed for comparison of continuous variables, and chi-square test for categorical variables. A two-tailed *P* < 0.05 was considered statistically significant.

## Results

### Study participants and demographic characteristics

A total of 428 cases with positive CSF cultures were retrospectively reviewed, including infections by *A. baumannii* (*n* = 145, 33.88%), *Klebsiella pneumonia* (*n* = 81, 18.93%), *Staphylococcus epidermidis* (*n* = 33, 7.71%), *Staphylococcus aureus* (*n* = 16, 3.74%), *Pseudomonas aeruginosa* (*n* = 11, 2.57%), and others such as *Klebsiella oxytoca*, *Cryptococcus neoformans*. Among the 145 cases with *A. baumannii-positive* CSF cultures, 84 subjects were excluded, and a total of 61 patients with intracranial infection due to MDR/XDR-Ab after neurosurgery were finally enrolled. There were 38 cases in the intravenous group and 23 cases in the intrathecal/intracerebral group. The baseline characteristics of these patients according to the two groups are summarized in Table 1, and no significant differences were observed in characteristics including age, sex, underlying disease, surgical history, use of external CSF drainage tube, APACHE II score, or SOFA score.

### Susceptibility testing and antimicrobial therapies

The detailed testing of the susceptibility of MDR/XDR-Ab to different antibiotics in these patients with intracranial infection after neurosurgery is described in Table 2. Among the most common antibiotics, MDR/XDR-Ab was most resistant to carbapenems (resistance rate of 100% to imipenem and to meropenem), cephalosporins (98.38% resistant to cefazolin, 100% to ceftazidime, 100% to cefatriaxone, and 98.38% to cefepime), whereas in no cases was MDR/XDR-Ab resistant to polymyxins. No significant differences in the results of susceptibility testing were observed between the two groups*.*

**Table 2:** Susceptibility testing results for isolated *A. baumannii*

	MIC Break-point	Total	ITV group	ITV + ITC group	
	(mg/L)	(*n* = 61)	(*n* = 38)	(*n* = 23)	*P*
Antibiotic resistance (n, %)					0.402
Amikacin	*R* ≥ 32	25, 40.98%	12, 31.58%	13, 56.52%	
Tigecycline	*R* ≥ 8	3, 2.92%	2, 5.26%	1, 4.35%	
Carbapenems					
Imipenem	*R* ≥ 8	60, 98.36%	37, 97.36%	23, 100.00%	
Meropenem	*R* ≥ 8	60, 98.36%	37, 97.36%	23, 100.00%	
Cephalosporins					
Cefazolin	*R* ≥ 32	60, 98.36%	38, 100.00%	22, 95.65%	
Ceftazidime	*R* ≥ 32	60, 98.36%	37, 97.36%	23, 100.00%	
Cefatriaxone	*R* ≥ 64	61, 100.00%	38, 100.00%	23, 100.00%	
Cefepime	*R* ≥ 32	60, 98.36%	37, 97.36%	23, 100.00%	
Polymyxin B	*R* ≥ 4	0, 0.00%	0, 0.00%	0, 0.00%	

*ITV* intravenous, *ITV + ITC* intrathecal/intracerebral

The most common antimicrobial regime used for the initially empirical therapy was meropenem/imipenem plus vancomycin (efficacy of 37.70%), followed by meropenem/imipenem only (18.03%), tigecycline plus cefperazone-sulbactam (16.39%), and meropenem/imipenem plus linezolid (14.75%; Table 3). Empirical antimicrobial use did not differ significantly between the two groups before intracranial infection (*P* = 0.684; Table 3). After MDR/XDR-Ab infection was confirmed, the most commonly employed antimicrobial regimes shifted to the combination of tigecycline plus cefperazone-sulbactam (31.15%), meropenem/imipenem plus tigecycline (19.67%), meropenem/ imipenem alone (18.03%) or cefperazone-sulbactam alone (13.11%) in the intravenous group.

**Table 3:** The initially applied empirical antimicrobial therapies

	Total (*n* = 61)	ITV group (*n* = 38)	ITV + ITC group (*n* = 23)	*P*
Before infection (n, %)				0.684
M/I + vancomycin	23, 37.70%	15, 39.47%	8, 34.78%	
M/I + linezolid	9, 14.75%	7, 18.42%	2, 8.70%	
M/I + cefperazone-sulbactam	2, 3.28%	2, 5.26%	0, 0.00%	
M/I	11, 18.03%	7, 18.42%	4, 17.39%	
Tigecycline + cefperazone-sulbactam	10, 16.39%	6, 15.78%	4, 17.39%	
Tigecycline	2, 3.28%	1, 2.63%	1, 4.34%	
Ceftriaxone	4, 6.56%	4, 10.53%	0, 0.00%	
Cefperazone-sulbactam + vancomycin	1, 1.64%	1, 2.63%	0, 0.00%	
Cefperazone-sulbactam	3, 4.92%	3, 7.89%	3, 13.04%	
Piperacillin-tazobactam	6, 9.84%	6, 15.79%	7, 30.43%	
After infection (n, %)				0.723
M/I + amikacin	3, 4.92%	3, 7.89%	0, 0.00%	
M/I + tigecycline	12, 19.67%	8, 21.05%	4, 17.39%	
M/I + cefperazone-sulbactam	7, 11.48%	4, 10.52%	3, 13.04%	
M/I	11, 18.03%	8, 21.05%	3, 13.04%	
Tigecycline+cefperazone-sulbactam	19, 31.15%	10, 6.32%	9, 39.13%	
Cefperazone-sulbactam	8, 13.11%	4, 10.53%	4, 17.39%	
Cefperazone-sulbactam + amikacin	1, 1.64%	1, 2.63%	0, 0.00%	

*ITV* intravenous, *ITV + ITC* intrathecal/intracerebral, /I meropenem/imipenem

### Microbiological clearance and biochemistry indicators of CSF

**Fig. 2:**
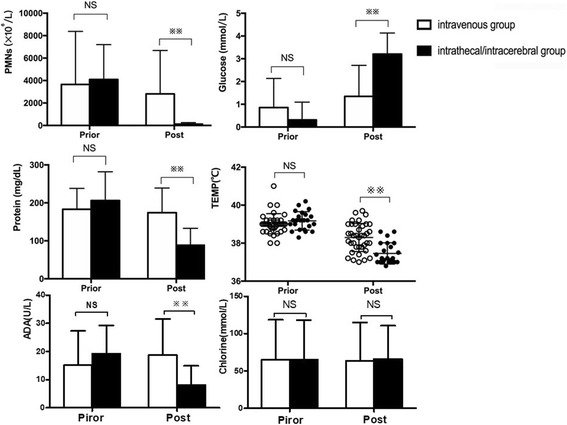
Laboratory indicators for CSF and body temperature before and after treatment in the two groups. PMNs: polymorph nuclear neutrophils; TEMP: temperature. ADA: adenosine deaminase. NS: not significant; **P < 0.01

The intrathecal/intracerebral group achieved a significantly higher microbiological clearance rate (91.30%, 21/23) than in the intravenous group (18.42%, 7/38; *P <* 0.01). Before treatment, there were no significant differences in the nuclei counts, chlorine, glucose, adenosine deaminase (ADA), and protein levels in CSF between the two groups. In comparison with the intravenous group, the intrathecal/intracerebral group showed a significantly decreased body temperature (39.20 ± 0.48 °C vs. 37.48 ± 0.56 °C, *P* < 0.01), a reduced number of nucleated cells in the CSF (4242.82 ± 3100.17 vs. 106.45 ± 120.00 × 10^6^/L, *P* < 0.01), greater recovery of the glucose level in CSF (0.56 ± 1.27 mmol/L vs. 3.20 ± 0.95 mmol/L, *P* < 0.01), a decreased level of ADA in the CSF (19.18 ± 10.02 U/L vs. 8.18 ± 6.78 U/L, *P* < 0.001), and reduced levels of total protein in CSF (207.10 ± 77.40 mg/dL vs. 87.81 ± 45.47 mg/dL, *P* = 0.012; Fig. 2).

### Clinical outcomes

Among the 61 patients with *A. baumannii* infection, the earliest death occurred on day two, and the total mortality rate was 37.70%. In the intravenous group, the mortality rate was 55.26% (21/38), while in the intrathecal/intracerebral group, the mortality rate was 8.70% (2/23; *P* = 0.01).

### Safety analysis

As renal function impairment is one of the side effects of polymyxin B treatment, we analyzed the changes in serum creatinine from before to after polymyxin B treatment. The mean creatinine level was 41.09 ± 11.46 μmol/L at 48 h after polymyxin B injection, which did not differ significantly from the baseline level (41.09 ± 11.46 μmol/L; *P* = 0.799).

## Discussion

*A. baumannii* is an opportunistic pathogen. The CHINET surveillance of bacterial resistance (2005–2014) [15] reported that *A. baumannii* accounts for 8.7–12.1% of clinical isolates in China, with the total number of bacterial isolates was ranging from 22,774–84,572 annually. In the current study, *A. baumannii* accounted for 33.88% of all isolates, which was considerably higher than the frequency noted in the 2005–2014 CHINET surveillance report. The reasons might be as follows: first, the incidence of *A. baumannii* infection is increasing; second, most of the patients recovering from neurosurgery were in an immune compromised state. Some had acquired artificial devices such as an external ventricular drain or intraventricular catheter, and some had hospitalized for a long time and had already received broad-spectrum antibiotics. All of these conditions are known risk factors for developing *A. baumannii* infection [16].

*A. baumannii* tends to quickly develop resistance to multiple antimicrobial agents through various mechanisms, such as through degrading enzymes targeting β-lactams, modifying enzymes targeting aminoglycosides, and alteration to the binding sites for quinolones [17]. A report from the SENTRY antimicrobial surveillance program (2001–2004) [18] showed that the resistance rates of *A. baumannii* exceeded 25% for imipenem and meropenem, 40% for cefepime and ceftazidime, 35% for amikacin, and 45% for ciprofloxacin. The CHINET surveillance report (2005–2014) from China [15] showed the resistance rate of *A. baumannii* for imipenem approximately doubled from 31% in 2005 to 62.4% in 2014, while that for meropenem increased from 39% in 2005 to 66.7% in 2014. Cefepime and ceftazidime resistance levels ranged from 54.8%–67.6% and 52.4%–71.9%, respectively, and amikacin and ciprofloxacin resistance levels ranged from 40.2%–61% and 60%–68.3%, respectively. In the present study, we found that 49.36% of *A. baumannii* isolates were MDR and 28.47% were XDR, with resistance to carbapenems (resistance rate of 100% for both imipenem and meropenem), cephalosporins (98.38% for cefazolin, 100% for ceftazidime, 100% for cefatriaxone, and 98.38% for cefepime). In comparison to the findings of the SENTRY antimicrobial surveillance report (2001–2004) and CHINET surveillance report (2005–2014), we observed major increases in the resistance of *A. baumannii* to these antibiotics. These results suggest that the treatment of *A. baumannii* is becoming more complicated, especially in the intensive care unit (ICU) where broad spectrum antimicrobials are commonly used.

For the initial empirical antimicrobial therapy, carbapenems, as broad-spectrum β-lactam antibiotics, have remained the first-line agents for patients who are immunocompromised or have had a prolonged period of hospitalization even though the prevalence of carbapenem-resistant bacteria is increasing [11]. Meropenem/imipenem is active against carbapenemase-negative *A. baumannii* isolates, but inactive against *A. baumannii* isolates that express plasmid-mediated carbapenemases [19]. Sulbactam is a β-lactamase inhibitor with intrinsic antibacterial activity against many *Acinectobacter* isolates, which is related to its affinity for penicillin-binding proteins [20]. It can be effective against infections caused by moderately imipenem-resistant isolates [21]. Because the combination of sulbactam and carbapenems showed better results than carbapenems alone for MDR-Ab infections [22], the combination of meropenem/imipenem and other antibiotics have been used as initial empirical therapy in our hospital. Considering that polymyxins are expensive and not easily accessible in China, after confirmation of MDR/XDR-Ab, most of the patients in the intravenous group received tigecycline together with cefperazone-sulbactam or meropenem/imipenem, but this did not effectively reduce mortality, which we attribute to the weak dispersion of tigecycline in CSF [23].

Consistent with the results of the CHINET report [15], the results of the present study demonstrate that *A. baumannii* is highly sensitive to polymyxins (100.00%), tigecycline (60.66%), and amikacin (49.18%). Thus, polymyxins may be an ideal antibiotic for the treatment of MDR/XDR-Ab, as they effectively and rapidly kill most Gram-negative microorganisms. The 2017 Infectious Diseases Society of America (IDSA) Clinical Practice Guide recommends that colistin or polymyxin B be administered intravenously and intraventricularly for the treatment of intracranial infections caused by carbapenem-resistant *Acinetobacter* species [11], but the quality of evidence is moderate as is mentioned in the Guide. In a retrospective study, Moon et al. [24] found that colistimethate-containing regimens could cure post-neurosurgical meningitis caused by carbapenem-resistant *A. baumannii*. Fotakopoulos et al. [25] reported that the combination of intravenous and intraventricular colistin may improve outcomes in patients with meningitis/ventriculitis due to multidrug resistance infections, especially that attributed to *A. baumannii*. Guardado et al. [6] studied intracranial infections caused by MDR/XDR-Ab after neurosurgery and found that intravenous injection along with intrathecal/intraventricular injection of polymyxin resulted in a significant reduction in mortality [0 vs. 80%, *P* = 0.04, odds ratio [OR]: 1.69 (1.32–2.16)]. Although a satisfactory result was achieved by intrathecal/intracerebral ventricle injection of polymyxins, the evidence remains rather weak as it stems from clinical studies or case series with small sample numbers. To our knowledge, our current study is the largest cohort study to date to compare the efficacy of intravenous combined with intrathecal/intracerebral ventricle injection of polymyxin B for intracranial infection due to MDR/XDR-Ab in post-neurosurgical patients. The intrathecal/intraventricular group showed significant improvement in microbiological eradication, biochemistry indicators of CSF, clinical efficiency, and 28-day mortality compared with the intravenous group. A retrospective case-control study analyzed the efficacy of intravenous plus intrathecal injection of colistin in 18 cases with XDR-Ab meningitis in the past 11 years [9]. It showed that the CSF sterilization rate was only 33.3% after treatment with intravenous administration of colistin alone, but reached complete sterilization with a rate of 100% after combination treatment with intravenous plus intraventricular injection of colistin (*P* = 0.009). These sterilization rates were similar to ours calculated in the present study. In Karaiskos’ study [26], the all-cause mortality rate of patients with intracranial infection caused by MDR/XDR-Ab was 71%, while the all-cause mortality rate in our study was only 47.54% (29/61). This could partially explain why intrathecal/intraventricular injection of polymyxin B significantly improved the survival rate, although it is impossible to discern the definitive cause of mortality. Evidence has revealed that the level of colistin in CSF is only 5–10% of that in blood when using intravenous administration only [27]. Moreover, the administration of polymyxin with direct intrathecal/intraventricular injection could increase the penetration of polymyxins into the central nervous system. Together with our study, all these findings suggest that a combination treatment with intravenous and intrathecal/intraventricular polymyxins be superior to routine intravenous antibiotics for the treatment of patients with an intracranial infection due to MDR/XDR-Ab.

A last-line treatment for infections that are resistant to other available antibiotics, the polymyxins antibiotics (including colistin and polymyxin B) are potentially nephrotoxic, but the relative risk of this adverse effect is still unclear [28]. Early reports revealed that use of more than the recommended dosage of colistin (2.5–5 mg/kg/day) was associated with an adverse renal reaction [29–31]. Additional research suggested that the incidence of nephrotoxic effects is higher with colistimethate than with polymyxin B [32]. In the present study, 61 patients received polymyxin B at a dose of 450,000 units per 12 h intravenously and at the same time received intrathecal/intraventricular injection of polymyxin B at 50,000 units/day. No cases of acute kidney injury were observed among the study participants according to the KDIGO guidelines [33], and several studies have shown that intravenous polymyxins is not associated with serious renal toxicity if the dosage is proper [34, 35]. Thus, the method of polymyxin B administration in the current study might be relatively safe for renal function. Notably, since the kidney is the primary route of elimination for polymyxins, the dosage must be carefully monitored [36].

Intravenous along with intrathecal/intracerebral injection of polymyxins might not only be effective for MDR/XDR-Ab but also have significant effects on other multidrug-resistant gram-negative bacteria. Macedo et al. [37] reported that intraventricular therapy with polymyxins improved outcomes in patients presenting with meningoencephalitis due to multidrug-resistant Gram-negative bacteria infections (*A. baumannii, P. aeruginosa,* etc.), with no cases of neurotoxicity and nephrotoxicity. In the study by Falagas et al. [35], intraventricular and intrathecal polymyxins (alone or with systemic antibiotics) were effective for Gram-negative meningitis *(P. aeruginosa, A. baumannii, etc),* and that toxicity is not uncommon but is usually dose-dependent and reversible. Therefore, taken together, these results suggest that intrathecal/intracerebral of polymyxins is an effective treatment strategy against intracranial infection by MDR/XDR-Ab or other MDR/XDR gram-negative bacteria without toxicity.

There were some limitations in this study. (1) This was a single-center retrospective study, and thus, further multicenter randomized controlled studies (prospective or retrospective) are needed. (2) The sample size in our current study (*n* = 61) was still small and needs to be expanded. (3) The most significant adverse effects of intraventricular or intrathecal injection that have been reported are chemical ventriculitis and meningitis. This study did not evaluate the neurotoxicity of polymyxin B nor obtain any dynamic records on changes in consciousness (such as the Glasgow Coma Scale score) and other clinical indicators due to the retrospective nature of the study. (4) Polymyxins must be bought from outside China, and they are too expensive for some patients.

## Conclusions

Intravenous plus intrathecal/intraventricular injection of polymyxin B can effectively improve levels of CSF indicators and support clinical efficiency, microbiologic eradication, and 28-day mortality without adverse effects, which might be a promising strategy to treat intracranial infections due to MDR/XDR-Ab.
