# Correction to: Efficacy of intravenous plus intrathecal/intracerebral ventricle injection of polymyxin B for post-neurosurgical intracranial infections due to MDR/XDR *Acinectobacter baumannii*: a retrospective cohort study

**DOI:** 10.1186/s13756-019-0462-1

**Published:** 2019-01-11

**Authors:** Sijun Pan, Xiaofang Huang, Yesong Wang, Li Li, Changyun Zhao, Zhongxiang Yao, Wei Cui, Gensheng Zhang

**Affiliations:** 1 Department of Critical Care Medicine, Second Affiliated Hospital, Zhejiang University School of Medicine, Hangzhou, Zhejiang 310009 People’s Republic of China; 2 Department of Critical Care Medicine, Anji County People’s Hospital, Huzhou, 313300 Zhejiang Province China; 3 Department of Critical Care Medicine, Zhejiang Hospital, Hangzhou, 310013 China

**Correction to: Antimicrob Resist Infect Control**

**https://doi.org/10.1186/s13756-018-0305-5**

The original article [1] contains an error in the **Safety analysis** sub-section of the results regarding the reported mean creatinine level. The correct mean creatinine level was 42.09 ± 18.54 μmol/L at 48 hours after polymyxin B injection instead of the reported 41.09 ± 11.46 μmol/L.
